# NLRP3 inflammasome-mediated choroid plexus hypersecretion contributes to hydrocephalus after intraventricular hemorrhage via phosphorylated NKCC1 channels

**DOI:** 10.1186/s12974-022-02530-x

**Published:** 2022-06-21

**Authors:** Zhaoqi Zhang, Qiang Tan, Peiwen Guo, Suna Huang, Zhengcai Jia, Xin Liu, Hua Feng, Yujie Chen

**Affiliations:** 1 Department of Neurosurgery and State Key Laboratory of Trauma, Burn and Combined Injury, Southwest Hospital, Third Military Medical University (Army Medical University), 29 Gaotanyan Street, Shapingba District, Chongqing, 400038 China; 2 Chongqing Key Laboratory of Precision Neuromedicine and Neuroregenaration, Southwest Hospital, Third Military Medical University (Army Medical University), Chongqing, 400038 China; 3 Chongqing Clinical Research Center for Neurosurgery, Southwest Hospital, Third Military Medical University (Army Medical University), Chongqing, 400038 China; 4 CAS Key Laboratory of Separation Science for Analytical Chemistry, Dalian Institute of Chemical Physics, Chinese Academy of Sciences, Dalian, 116023 China

**Keywords:** Intraventricular hemorrhage, Hydrocephalus, NLRP3 inflammasome, Choroid plexus, NKCC1, Cerebrospinal fluid hypersecretion

## Abstract

**Background:**

Hydrocephalus is a severe complication of intracerebral hemorrhage with ventricular extension (ICH-IVH) and causes cerebrospinal fluid (CSF) accumulation. The choroid plexus epithelium plays an important role in CSF secretion and constitutes the blood–CSF barrier within the brain–immune system interface. Although the NLRP3 inflammasome, as a key component of the innate immune system, promotes neuroinflammation, its role in the pathogenesis of hydrocephalus after hemorrhage has not been investigated. Therefore, this study aimed to investigate the potential mechanism of NLRP3 in hydrocephalus to discover a potential marker for targeted therapy.

**Methods:**

A rat model of hydrocephalus after ICH-IVH was developed through autologous blood infusion in wild-type and Nlrp3^−/−^ rats. By studying the features and processes of the model, we investigated the relationship between the NLRP3 inflammasome and CSF hypersecretion in the choroid plexus.

**Results:**

The ICH-IVH model rats showed ventricular dilation accompanied by CSF hypersecretion for 3 days. Based on the choroid plexus RNA-seq and proteomics results, we found that an inflammatory response was activated. The NLRP3 inflammasome was investigated, and the expression levels of NLRP3 inflammasome components reached a peak at 3 days after ICH-IVH. Inhibition of NLRP3 by an MCC950 inflammasome inhibitor or Nlrp3 knockout decreased CSF secretion and ventricular dilation and attenuated neurological deficits after ICH-IVH. The mechanism underlying the neuroprotective effects of NLRP3 inhibition involved decreased phosphorylation of NKCC1, which is a major protein that regulates CSF secretion by altering Na^+^- and K^+^-coupled water transport, via MCC950 or Nlrp3 knockout. In combination with the in vitro experiments, this experiment confirmed the involvement of the NLRP3/p-NKCC1 pathway and Na^+^ and K^+^ flux.

**Conclusions:**

This study demonstrates that NKCC1 phosphorylation in the choroid plexus epithelium promotes NLRP3 inflammasome-mediated CSF hypersecretion and that NLRP3 plays an important role in the pathogenesis of hydrocephalus after hemorrhage. These findings provide a new therapeutic strategy for treating hydrocephalus.

**Supplementary Information:**

The online version contains supplementary material available at 10.1186/s12974-022-02530-x.

## Background

Intracerebral hemorrhage (ICH) occurs in 10–15% of all strokes worldwide each year [1]. Of the approximately 40% of ICH cases that develop intraventricular hemorrhage, 50% are complicated by hydrocephalus [2, 3]. Extension of the hemorrhage into the ventricular system after ICH leads to hydrocephalus, which is the accumulation of cerebrospinal fluid (CSF) in the ventricles [4]. Persistent elevations in intracranial pressure can cause acute brainstem herniation and, ultimately, death [5]. Invasive CSF shunting is the first line of treatment for hydrocephalus after hemorrhage, but it carries a high risk for serious complications, such as shunt obstruction or infection [6, 7]. Therefore, it is necessary to develop fast-acting, low-risk targeted pharmacotherapeutic strategies for treating patients with hydrocephalus [8].

CSF circulation failure is the major mechanism of hydrocephalus after hemorrhage. Most relevant studies have aimed to develop techniques for controlling the flow of CSF by targeting the obstruction. Hydrocephalus after hemorrhage becomes aggravated by damage to ependymal glia and dysfunction of arachnoid granulations, which can block the outflow of CSF [9, 10]. The Toll-like receptor 4 (TLR4) protein-dependent inflammatory response contributes to hydrocephalus by inducing hypersecretion of CSF [11]. CSF is predominantly produced by the choroid plexus, which is an epithelial monolayer that serves as the main component of the blood–CSF barrier [12, 13]. The Na^+^/K^+^/2Cl^−^ cotransporter (NKCC1), which is expressed in the luminal membrane of the choroid plexus, contributes to approximately half of the production of CSF [14]. NKCC1 contributed to CSF formation by transporting Na^+^, K^+^, and Cl^−^ transmembrane coupled water, which permits water to be transported independently of, and even against, an osmotic gradient [15]. As a chloride importer antagonist of NKCC1, bumetanide attenuates many neurological and psychiatric disorders [16]. Intracerebroventricular injection of blood metabolites caused choroid plexus inflammation and hydrocephalus, but the mechanisms remain unclear. Secretory epithelia respond to proinflammatory stimulation by increasing the fluid secretion rate.

As a critical function of the innate immune response to tissue injury, the NLRP3 inflammasome is increasingly being considered to be a driver of injury after hemorrhage; this inflammasome senses cellular deviation from homeostasis as a danger signal and subsequently initiates inflammatory responses [17–19]. The NLRP3 inflammasome releases cytokines (IL-1β and IL-18) and exacerbates brain edema, disruption of the blood–CSF barrier, and neuronal apoptosis after ICH [20, 21]. Furthermore, when NLRP3 was suppressed (MCC950 or Nlrp3^−/−^), the secondary brain injury caused by ICH was alleviated, and most relevant studies have aimed to investigate the NLRP3 inflammasome in immune cells, such as microglia, macrophages and astrocytes [22, 23]. However, no functional or regulatory interactions have been demonstrated in choroid plexus epithelial cells to date.

Despite partial elucidation of the underlying mechanism of hydrocephalus after hemorrhage, its pathogenesis is not well understood. To achieve a comprehensive understanding of the biological processes of this difficult-to-treat condition, based on transcriptomics and proteomics of the choroid plexus, we hypothesized that NLRP3 would aggravate hydrocephalus after ICH with ventricular extension (ICH-IVH) by altering NKCC1 phosphorylation to enhance CSF secretion in the choroid plexus. The present study investigated the function and molecular mechanism of the NLRP3 inflammasome in the pathogenesis of hydrocephalus with the aim of discovering a new therapeutic target for hydrocephalus patients.

## Methods

### Animals

Adult male Sprague-Dawley rats (220–250 g) were purchased from the Animal Experimental Center of Third Military Medical University (Chongqing, China). *Nlrp3*^−/−^ rats were purchased from Cyagen Biosciences (Guangzhou, China). The rats were housed in a temperature-controlled room under specific pathogen-free conditions and a standard 12-h light/dark cycle, with ad libitum access to food and water. All experimental procedures involving animals were performed in compliance with the Guidelines for the Care and Use of Laboratory Animals, approved by the Laboratory Animal Welfare and Ethics Committee of Third Military Medical University (AMUWEC2020762), and reported according to the ARRIVE (Animal Research: Reporting of In Vivo Experiments) guideline.

### ICH-IVH and IVH models of hydrocephalus and drug treatment

**Fig. 1:**
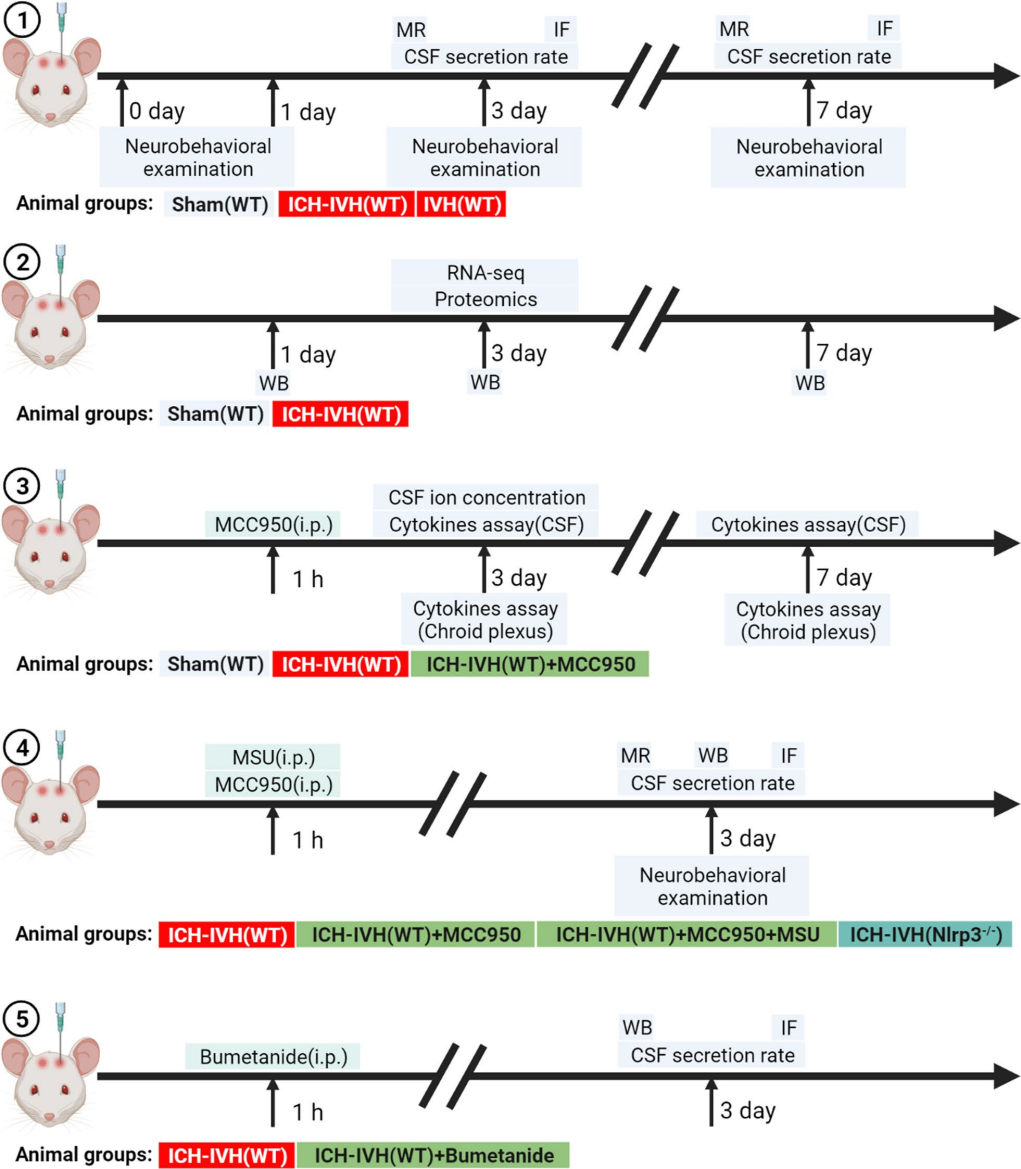
Schematic experimental design and subsequent analyses. MR indicates T2-weighted images; IF indicates immunofluorescence staining; i.p. indicates intraperitoneal injection

The surgical procedures used in this study for rats subjected to hemorrhage followed the procedures that are described in detail in our previous study [9, 24]. In brief, the animals were anesthetized with pentobarbital (40 mg/kg, intraperitoneally). The right femoral artery was catheterized as the source for obtaining a blood sample. For the ICH-IVH model, the rats were positioned in a stereotaxic frame, in which a cranial burr hole (1 mm) was drilled (coordinates: 0.2 mm posterior and 2.2 mm lateral to bregma). A 29-gauge needle was inserted at a rate of 1 mm/min at a depth of 5.0 mm from the dura. Using a microinjection pump, 200 μl of nonheparinized arterial blood was infused through the hole into the right caudate nucleus at a rate of 14 μl/min. In the IVH model, only the location of the drilled hole, which was drilled at 0.6 mm posterior and 2.2 mm lateral to the bregma, was different from that in the ICH-IVH model, and the depth of the inserted needle was 4.5 mm. After ICH-IVH, all rats were randomly divided into 4 groups as follows: ICH-IVH, ICH-IVH + MCC950, ICH-IVH + MCC950 + MSU, and ICH-IVH + bumetanide (Fig. 1). The process of randomization was as follows: all ICH-IVH rats were numbered and separated into four groups according to automatic random number blind grouping. The NLRP3 inhibitor MCC950 (MCE, USA) dissolved in saline was administered at a dose of 10 mg/kg by intraperitoneal injection 1 h after ICH-IVH induction. The NLRP3 activator monosodium urate (Abcam, USA), dissolved in saline, was administered by intraperitoneal injection after the MCC950 injection. Bumetanide (MCE, USA) dissolved in saline was administered at a dose of 10 mg/kg by intraperitoneal injection each day after ICH-IVH induction. The ICH-IVH group was given an equal volume of normal saline at the same time. The sham group received only needle injections following the ICH-IVH rats.

### Neurobehavioral examination

#### Modified Neurological Severity Score (mNSS)

The rats were evaluated for neurological dysfunction using the mNSS method, as described previously [25]. The mNSS is the result of a composite test of motor, sensory and balance functions. Briefly, the assessment was performed at 3 days and 7 days after ICH-IVH. Neurological function was graded on a scale of 0–18 (a score of 13–18 indicates severe injury, 7–12 indicates moderate injury, and 1–6 indicates mild injury).

#### Rotating beam test

A rotarod test was performed to evaluate sensorimotor deficits as previously described [26]. Briefly, animals were placed on a rotarod cylinder (RWD, China). The time until the animals fell off the rotating cylinder was recorded.

#### Grip front test

The grip strength of the front limbs was measured by a grip strength meter (Laboratory Enterprises, Nasik, India), which consisted of a steel wire grid connected to an isometric force transducer following a method described earlier [27]. Each rat was measured in triplicate, and the average handgrip strength of each was recorded. The rats were gently pulled back when they grasped the grid with their forepaws until they released the grid, and the maximal force in Newtons exerted by the rats before they lost their grip was measured.

All behavioral tests were performed by an investigator who was blinded to the treatments.

### Quantification of the CSF secretion rate

The rates of CSF production were measured using a method as previously described [28]. Briefly, anesthetized rats were mounted on a stereotactic apparatus, and a cranial burr hole (1.2 mm) was drilled over the left lateral ventricle (coordinates relative to bregma: *x*, − 0.6 cm; *y*, − 1.6 cm). Next, the rat’s head was rotated on the ear bars by 90° so that it was oriented nose down, and the suboccipital muscles were dissected to the cisterna magna to expose the atlantooccipital ligament. The ligament was punctured, and a 29-gauge needle was advanced 5 mm through the foramen of Magendie to the fourth ventricle. Sterile molecular-grade oil (100 μl; Sigma-Aldrich) was infused through the tube to occlude the aqueduct of Sylvius, thereby creating a closed system of CSF circulation in the lateral ventricle. With the rat in the same position, a glass capillary tube (OD, 1.1 mm; ID, 1.0 mm; length, 20 cm) was advanced through the burr hole into the lateral ventricle (depth 4.5 mm ventral). The volume of CSF collected at a given time (20 min) was calculated as *V* (mm^3^) = π·*r*^2^·*d*, where *r* is the radius of the glass capillary tube and *d* is the distance that CSF traveled within the capillary tube. The rate of CSF formation (μl/min) was then calculated from the slope of the volume–time relationship.

### Western blot analysis

Western blot analysis was performed as previously described [29]. The brains were perfused with saline before decapitation at day 3 post-injection. The choroid plexus tissue was sampled. The following primary antibodies were used: rabbit anti-NLRP3 (1:1000 dilution, Abcam, ab210491, UK); mouse anti-Caspase-1 (1:1000 dilution, Novus, 56565SS, USA); rabbit anti-IL-1β (1:1000 dilution, Gene-Tex, 55675, USA); rabbit anti-p-NKCC1 (1:1000 dilution, Sigma-Aldrich, ABS1004, USA); rabbit anti-NKCC1 (1:1000 dilution, CST, 14581S, USA) and rabbit anti-β-actin (1:1000 dilution, CST, 8457, USA). The relative densities of the immunoreactive bands were normalized to that of β-actin and then analyzed using ImageJ (National Institutes of Health, Bethesda, Maryland, USA).

### Cytokine assay kits

Rats were anesthetized with pentobarbital (40 mg/kg intraperitoneal). CSF was collected through the cisterna magna; then, the rats were decapitated to obtain the choroid plexus. The CSF was centrifuged at 10,000 rpm to separate the supernatant. The choroid plexus was homogenized in ice-cold phosphate-buffered saline with protease inhibitor cocktails (Sigma-Aldrich, USA). Total protein concentration was quantified using a bicinchoninic acid protein assay (Boster, China). The CSF and choroid plexus cytokine levels were measured using inflammation cytokine assay kits (RayBiotech, USA) according to the manufacturer’s instructions.

### Immunofluorescence staining

Immunofluorescence staining of the brain tissue was performed on fixed frozen sections, as previously described [30]. Rats were anesthetized with pentobarbital (40 mg/kg intraperitoneal) and perfused with 4% paraformaldehyde after deep anesthesia was achieved. The brain was isolated, and then the choroid plexus was dissected under magnification using sharp forceps. The choroid plexus was subsequently immersed in 30% sucrose for 3–4 days at 4 °C. The tissues were embedded in an optimal cutting temperature compound (Sakura, USA), and 12-mm-thick slices were cut using a cryostat. The slices were incubated with the following primary antibodies at 4 °C overnight: a rabbit antibody to NLRP3 (1:200 dilution, ab4207, Abcam, UK), a rabbit antibody to laminin (1:100 dilution, Abcam, UK), a rabbit antibody to AE2 (1:200 dilution, ab42687, Abcam, UK), a goat antibody to NLRP3 (1:100, Gene-Tex, USA), a rabbit antibody to AE2 (1:200 dilution, Abcam, UK) and a rabbit antibody to p-NKCC1 (1:200 dilution, ABS1004, Sigma-Aldrich, USA). Then, the slices were probed with appropriate secondary antibodies for 2 h at 37 °C. Finally, the slices were counterstained with 4′,6-diamidino-2-phenylindole (Boster, China) and examined using a confocal fluorescence microscope (LSM780, Zeiss).

### Cell counts

Cells were counted 3 days after ICH-IVH. For quantification of the positive cells in the choroid plexus, consecutive slices were made in two sections per animal (*n* = 6 per group), with a 40-μm space in between used for cell counts. Three high-power images were used for cell counting. NLRP3- and p-NKCC1-positive cells were counted in the choroid plexus. Cell counts were performed by two researchers in a blinded manner. All measurements were repeated three times, and the mean values were used.

### MRI and ventricular volume analysis

Rats were anesthetized with a 2% isoflurane/air mixture throughout MRI examination. The MRI scans were performed using a 7.0-T Varian MR scanner (Bruker, USA) with a T2*gradient-echo sequence and a T2 fast spin-echo sequence using a view field of 35 mm × 35 mm and 17 coronal slices (1.0 mm thickness). There are two classical methods for evaluating hydrocephalus based on MRI, i.e., the Evans index and whole-ventricle volume assessments [31]. Considering the time window and goals of our experiments, the whole-ventricle volume was calculated as according to a previously described method [32]. Bilateral ventricles and the hippocampus were outlined, and the areas of all slices were multiplied by the section thickness [33]. The 3D reconstruction of ventricular systems was based on the 3D Slicer. All image analyses were performed using ImageJ (National Institutes of Health, Bethesda, Maryland, USA) by two observers in a blinded manner.

### CSF collection and metal detection

CSF was collected by inserting a syringe into the cisterna magna, and the collected CSF was centrifuged at 10,000×*g* for 5 min at 4 °C to remove any tissue debris. Inductively coupled plasma-optical emission spectrometry (ICP-OES) was used for K^+^ and Na^+^ quantification. All tests were performed with 5–10 μl of CSF.

### Transcriptome sequencing, quantitative proteomic and integrated analysis

#### Quantitative RNA sequencing

The rats in the sham group and ICH-IVH group (3 days) were anaesthetized on ice and dissected longitudinally to obtain the choroid plexus (*n* = 6 in each group, 3 replicates). Total RNA of the choroid plexus was isolated using TRIzol reagent (Invitrogen, USA). The quantity and purity of total RNA were then detected by a NanoDrop ND-1000 instrument (NanoDrop, USA). Furthermore, RNA integrity was detected by an Agilent 2100 instrument, and a RIN > 7.0 was taken as the qualified standard. Then, the qualified RNA was provided to LC-Bio Technology Co., Ltd. (Hangzhou city, China), for subsequent library preparation and sequencing using a HiSeq 4000 platform as described previously [34]. The expression levels of all transcripts were then evaluated by calculating the fragments per kilobase per million reads (FPKM). The thresholds of significantly different expression were *p* < 0.05 and FPKM > 1. The GO and KEGG databases were used to explore the functions and biological pathways in which the differentially expressed genes were involved.

#### Proteomics analysis

The choroid plexus was rapidly frozen and ground in liquid nitrogen and then treated as previously reported to obtain the purified protein concentration [35]. Briefly, TMT reagent (Thermo Fisher Scientific) was added to the prepared protein suspension to obtain a peptide mixture for sequencing by LC-Bio (http://www.lc-bio.com/). Differentially expressed proteins were defined as proteins with a fold-change greater than 1.2 and a *p* value (*t*-test) less than 0.05. Functional classification of differentially expressed proteins was conducted by mapping with GO terms, and the highest bit score sequence was selected. Pathway analysis was performed using the KEGG database.

### Primary choroid plexus epithelial cell cultures and stimulator administration

Primary choroid plexus epithelial cells from rats were cultured as previously described [36]. In brief, the brains of P14 rats were isolated, and the choroid plexus was isolated and plated into freshly prepared sterile dissection media. Dissection media included 1 × DPBS with 0.6% glucose and 1 × Pen-Strep. The tissue was then resuspended in a working solution (a mixture of 5 × collagenase [type II] and 1 × HBSS supplemented with 3 mM CaCl_2_ at a 1:5 ratio) and incubated at 37 °C for 20 min; the tubes were tapped every 5 min. CPEC media included 10% FBS and 10,000 units/ml Pen-Strep in DMEM. Tissue was dissociated using a pipette and then plated on poly-d-lysine and laminin-coated cell culture plasticware. Choroid plexus epithelial cells were cultured for 14 days, with the media changed every 2–3 days, and then used for experiments. Cells were treated with 1 μg/ml LPS or 1 μg/ml lysis-RBCs for 12 h. Thirty minutes after incubation with lysis-RBCs, MCC950 (5 μM) was added.

### Statistical analysis

The data were analyzed and plotted by GraphPad Prism and presented as the means ± SEMs. Sample sizes were calculated using an a priori sample size calculator with the following assumptions: *A* = 0.05; two-tailed; and desired power, 80%. The results indicated that a minimum of 3 rats per group were needed. All data were analyzed using GraphPad Prism and satisfied a normal distribution and the homogeneity of variance. Immunostaining, cytokine measurement data, and behavioral experiments were subjected to one-way ANOVA. Student’s *t*-test was used for single comparisons, and analysis of variance with post hoc Bonferroni–Dunn correction was used for multiple comparisons, depending on the experiments. A *p* value < 0.05 was considered to be statistically significant. The number of different experimental groups and statistical methods is shown in the figure legends, and statistical analysis data are detailed in the Additional file 1: Statistical results.

## Results

### ICH-IVH induced CSF hypersecretion and hydrocephalus and neurocognitive functional deficits

**Fig. 2:**
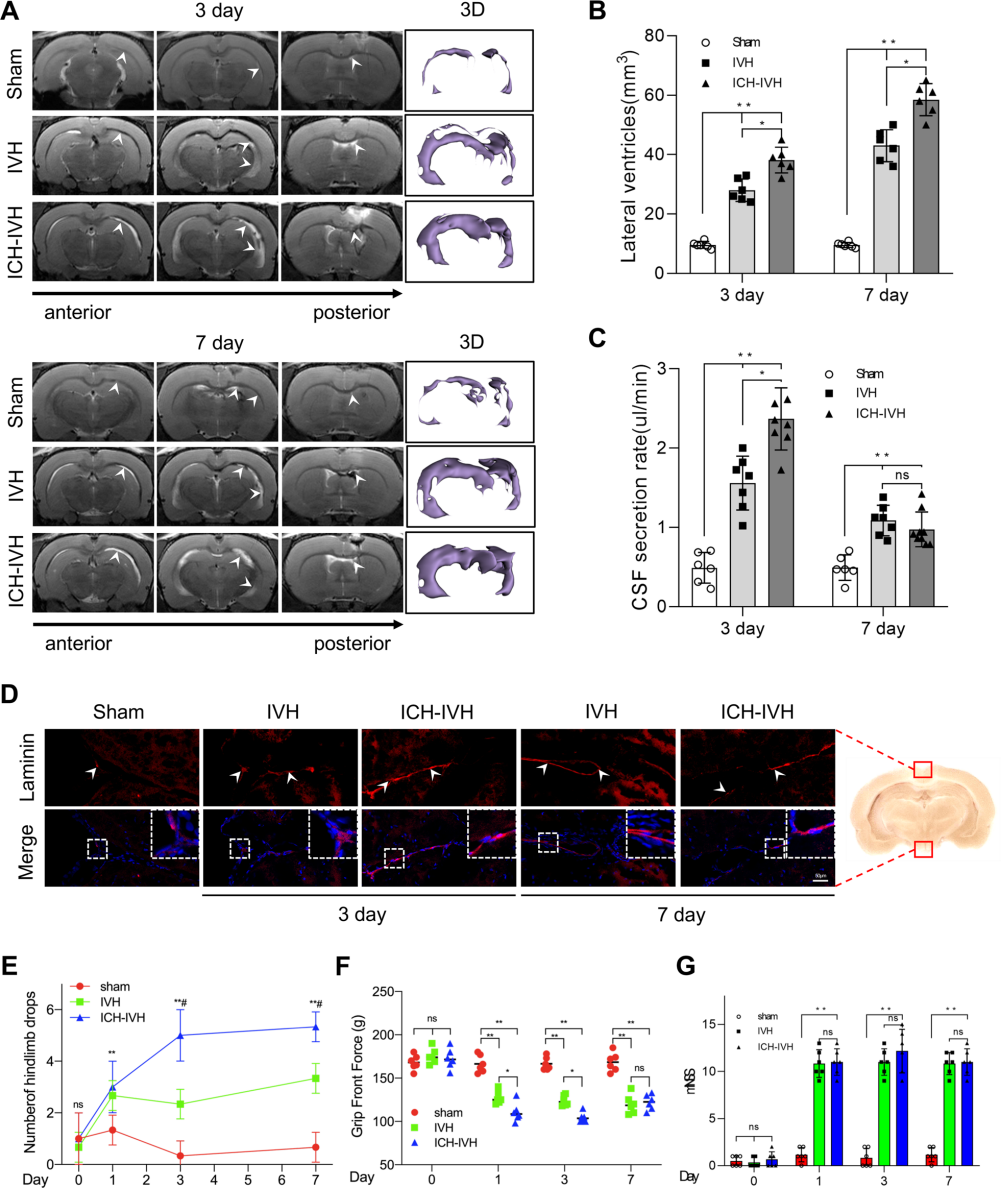
CSF secretion rate and hydrocephalus evaluation index after ICH-IVH and IVH. **A** Representative T2-weighted images and 3D reconstruction of lateral ventricles obtained on day 3 and day 7 in the IVH and ICH-IVH groups. **B** Quantification volumes of the lateral ventricle according to the related T2-weighted images (6 rats/group, one-way ANOVA). **C** Quantification of CSF secretion rates in sham, IVH and ICH-IVH rats at 3 and 7 days (6–8 rats/group, one-way ANOVA). **D** Representative images of immunofluorescence staining for laminin expression in arachnoid granules. Bar = 50 μm. **E** Number of hindlimb drops recorded for a walking task in which the rat walks over a rotating beam toward the home cage on a platform (6 rats/group, one-way ANOVA). **F** Testing for front grip strength at different time points (6 rats/group, one-way ANOVA). **E** mNSS (6 rats/group, one-way ANOVA). The results are presented as the means ± SDs; **p < 0.01 and *p < 0.05: sham group versus ICH-IVH group. #*p* < 0.05: sham group versus IVH group

According to a previous posthemorrhagic hydrocephalus animal model, intraventricular hemorrhage (IVH) and ICH-IVH rat models were constructed [9]. Both of them obviously involved hydrocephalus. We first evaluated the degree of hydrocephalus in the two different rat models by measuring lateral ventricular volumes according to T2 magnetic resonance imaging (MRI) scans combined with 3D reconstruction images at 3 and 7 days after hemorrhage (Fig. 2A). We found that ICH-IVH had more obvious lateral ventricular dilation than did IVH. Both of them already showed severe hydrocephalus 3 days after hemorrhage and worsened over time. Compared with the sham group, both hemorrhage groups had more obvious lateral ventricular volumes, which indicated hydrocephalus (Fig. 2B). The occurrence of hydrocephalus depends on CSF circulation failure, which involves two main aspects: CSF formation and outflow [37]. Laminin staining of arachnoid granules was performed to investigate whether the flowing-out deficits occurred at 3 days after hemorrhage. The images showed that there was no arachnoid granule fibrosis to influence the CSF outflow and contribute to hydrocephalus until 7 days after hemorrhage (Fig. 2D). Therefore, we speculated that more CSF produced by the choroid plexus mediated hydrocephalus during the acute phase of 3 days. Based on previous methods [28], we measured the CSF secretion rate in vivo and found that the CSF secretion rate was elevated after hemorrhage. Additionally, the ICH-IVH group had a higher CSF secretion rate than the IVH group did at both 3 days and 7 days. In addition, the CSF secretion rate decreased over time after hemorrhage and reached a peak at 3 days (Fig. 2C). To determine whether the high CSF secretion rate mediated acute-phase hydrocephalus and influenced the neurofunctions, we assessed the neurofunctions after hemorrhage. Hindlimb tests were used to evaluate the balance ability. We compared the number of hindlimb drops after hemorrhage and found that the hemorrhage groups experienced similar deficits, especially the ICH-IVH group (Fig. 2E). Next, we tested the grip front force and found that the grip front force weakened in the hemorrhage groups (Fig. 2F). The mNSS comprehensively evaluated neurofunctions and showed that the hemorrhage groups had severe neurological deficits (Fig. 2G). According to the neurofunction tests, we found hydrocephalus after hemorrhage in rats with neurodeficit. Thus, it can be concluded that the ICH-IVH group had more severe neurodeficit than the IVH group did. Taken together, these data confirmed that the ICH-IVH groups had obvious hydrocephalus and neurodeficit in the acute phase and that CSF secretion contributed to hydrocephalus in this phase.

### Quantitative transcriptome sequencing and proteomic analysis of the choroid plexus revealed inflammatory protein and RNA accumulation after ICH-IVH

**Fig. 3:**
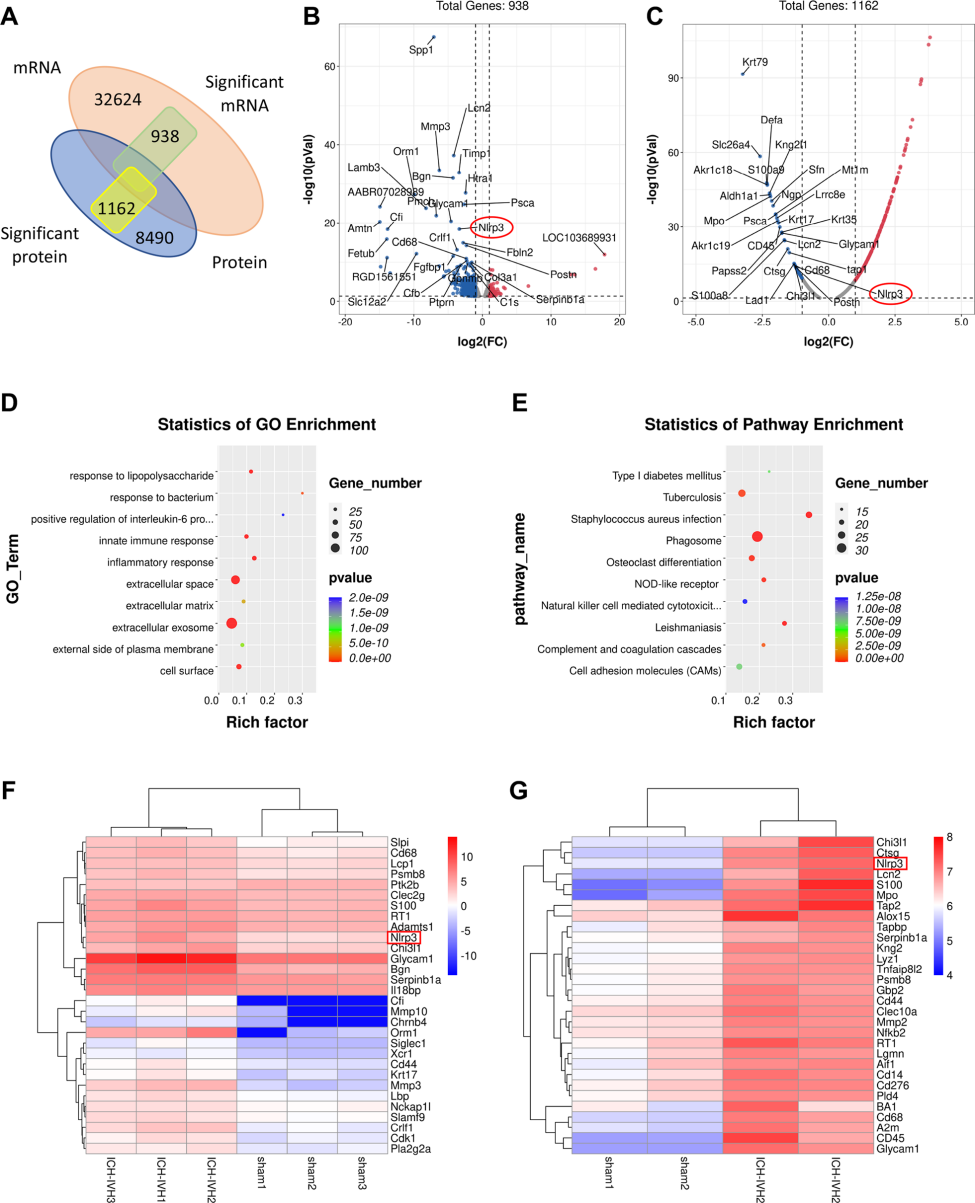
Transcriptome and proteomic sequencing was performed to explore inflammation pathways activated after ICH-IVH in the choroid plexus. **A** Venn diagram showing RNA and protein sequencing. **B** Volcano map showing significantly changed genes identified by transcriptome sequencing between the sham group and ICH-IVH group. **C** Volcano map showing significantly changed genes identified by proteomic sequencing after ICH-IVH. **D** GO enrichment of transcriptome sequencing showing that the inflammatory response was upregulated after ICH-IVH. **E** KEGG pathway enrichment of transcriptome sequencing showed that NOD-like receptors were upregulated after ICH-IVH. **F** Heatmap of transcriptome sequencing showing the top 30 genes related to inflammation between the sham group and ICH-IVH group. (H) Heatmap of proteomic sequencing showing the top 30 proteins related to inflammation after ICH-IVH

We dissected the brains of six pairs of sham and ICH-IVH rats to expose the choroid plexus and performed quantitative tandem mass spectrometry experiments using 10-plex tandem mass tag labeling. Transcriptome sequencing was performed on 32624 RNAs, and 938 RNAs were obviously changed after ICH-IVH. In total, we quantified 8490 proteins that overlapped in the two groups. Compared with the sham group, the ICH-IVH group had 1162 significantly changed proteins (*p* < 0.05). According to transcriptome and proteomic analysis of the choroid plexus, we found some change trends at the RNA and protein levels (Fig. 3A), indicating that our quantitative mass spectrometry method could differentiate the choroid plexus transcriptome and proteome by hemorrhage. A volcano map of transcriptome sequencing shows the significantly different genes, and the top 30 genes are marked, including some inflammation- and innate immunity-related genes (Fig. 3B). Proteomic analysis of the volcano map showed significantly different proteins, and we marked the top 30 obviously changed proteins (Fig. 3C). Furthermore, using GO enrichment and pathway enrichment to explore the functions of these genes, we found that the inflammatory response was obviously activated in the choroid plexus after hemorrhage (Fig. 3D, E). According to genes whose expression obviously changed at the RNA level, we marked the top 30 inflammation-related genes in the heatmap (Fig. 3F). A combined heatmap of proteomic analysis marked the top 30 proteins related to inflammation (Fig. 3G). The NLRP3 inflammasome was selected from inflammation-related genes according to transcriptome and proteomic analysis for further investigation. The NLRP3 inflammasome plays an important role in the CNS and contributes to many kinds of biological processes, including immune and inflammatory responses, and is highly expressed in macrophages, microglia, astrocytes, and epithelial cells. Studies on the NLRP3 inflammasome in epithelial cells are rare, especially in terms of choroid plexus epithelial cells. Therefore, we investigated the function of NLRP3 in hydrocephalus.

### Time course of NLRP3 inflammasome changes in the choroid plexus after ICH-IVH

**Fig. 4:**
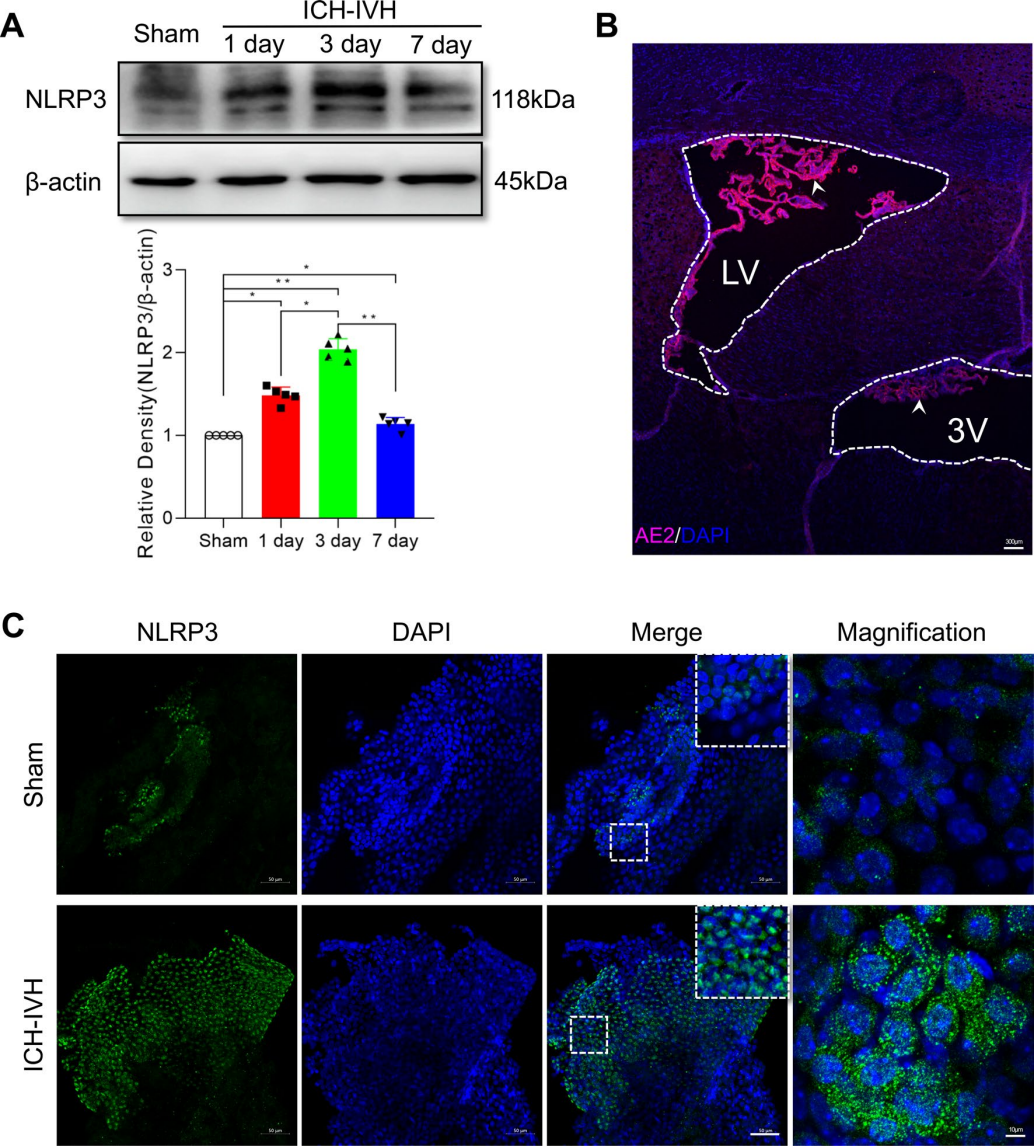
NLRP3 inflammasome components were activated after ICH-IVH in the choroid plexus. **A** Western blot analysis of NLRP3 in the choroid plexus over time and statistical analysis of NLRP3 protein levels achieved at a maximum of 3 days after ICH-IVH (*n* = 5, 6 rats/sample, one-way ANOVA). **B** Representative images showing the choroid plexus AE2 location in ventricular systems; LV: lateral ventricle; 3 V: the third ventricle; the white arrow indicates the choroid plexus. Bar = 300 μm. **C** Representative fluorescence photomicrographs showing immunolabeling for NLRP3-positive cells in the choroid plexus 3 days after ICH-IVH (6 rats/group). Bar = 50 μm. The values are expressed as the means ± SDs; **p < 0.01; *p < 0.05

**Fig. 5:**
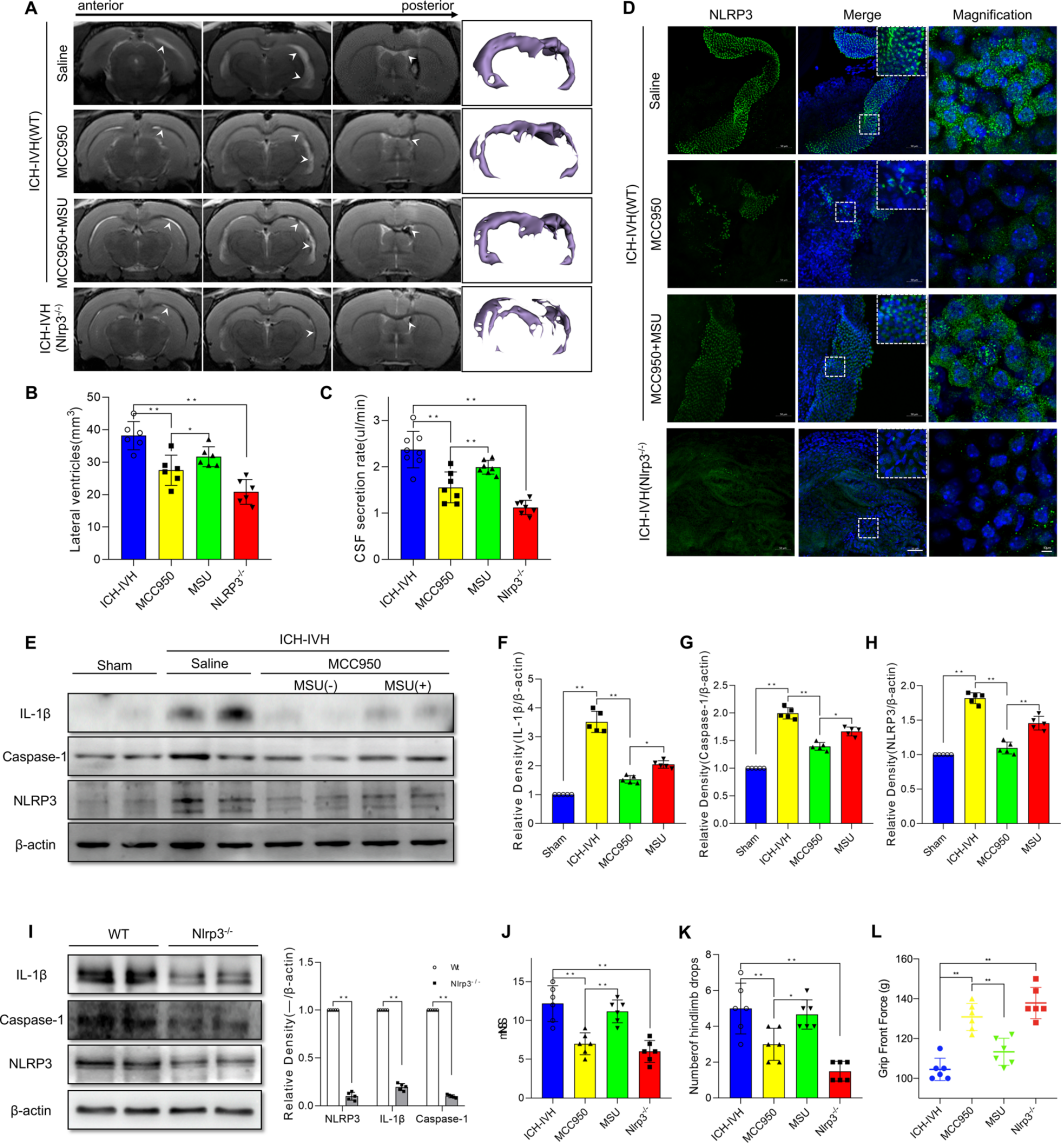
Inhibitor or knockout of NLRP3 decreased NLRP3-related inflammation, CSF secretion and hydrocephalus. **A**, **B** Representative T2-weighted images of brain tissue, 3D reconstruction of the lateral ventricle after different NLRP3 treatments (**A**) and lateral ventricle volumes (**B**) measured via T2-weighted images (6 rats/group, one-way ANOVA). **C** CSF secretion rate in the MCC950-inhibited NLRP3 and Nlrp3^−/−^ groups (6–8 rats/group, one-way ANOVA). **D** Representative images of immunofluorescence staining for NLRP3 expression in the choroid plexus 3 days after ICH-IVH and different treatments. Bar = 50 μm. **E**–**H** Western blot analysis of NLRP3, caspase-1, and IL-1β in the choroid plexus of ICH-IVH rats, MCC950-treated rats and MSU-treated rats on day 3 (*n* = 5, 6 rats/sample, one-way ANOVA). **I** Western blot analysis of NLRP3, caspase-1, and IL-1β in the choroid plexus after ICH-IVH in the control and Nlrp3^−/−^ groups (n = 5, 6 rats/sample, Welch’s two-tailed unpaired *t*-test). **J** mNSS (6 rats/group, one-way ANOVA). **K** Number of hindlimb drops recorded for a walking task in which the rat walks over a rotating beam toward the home cage on a platform (6 rats/group, one-way ANOVA). **L** Testing for front grip strength at different time points (6 rats/group, one-way ANOVA). **B**, **C**, **F**–**H**, **J**–**L** ICH-IVH indicates ICH-IVH (WT) + saline; MCC950 indicates ICH-IVH (WT) + MCC950; MSU indicates ICH-IVH (WT) + MCC950 + MSU; Nlrp3^−/−^ indicates ICH-IVH (Nlrp3^−/−^). The values are expressed as the means ± SDs; **p < 0.01 and *p < 0.05

**Fig. 6:**
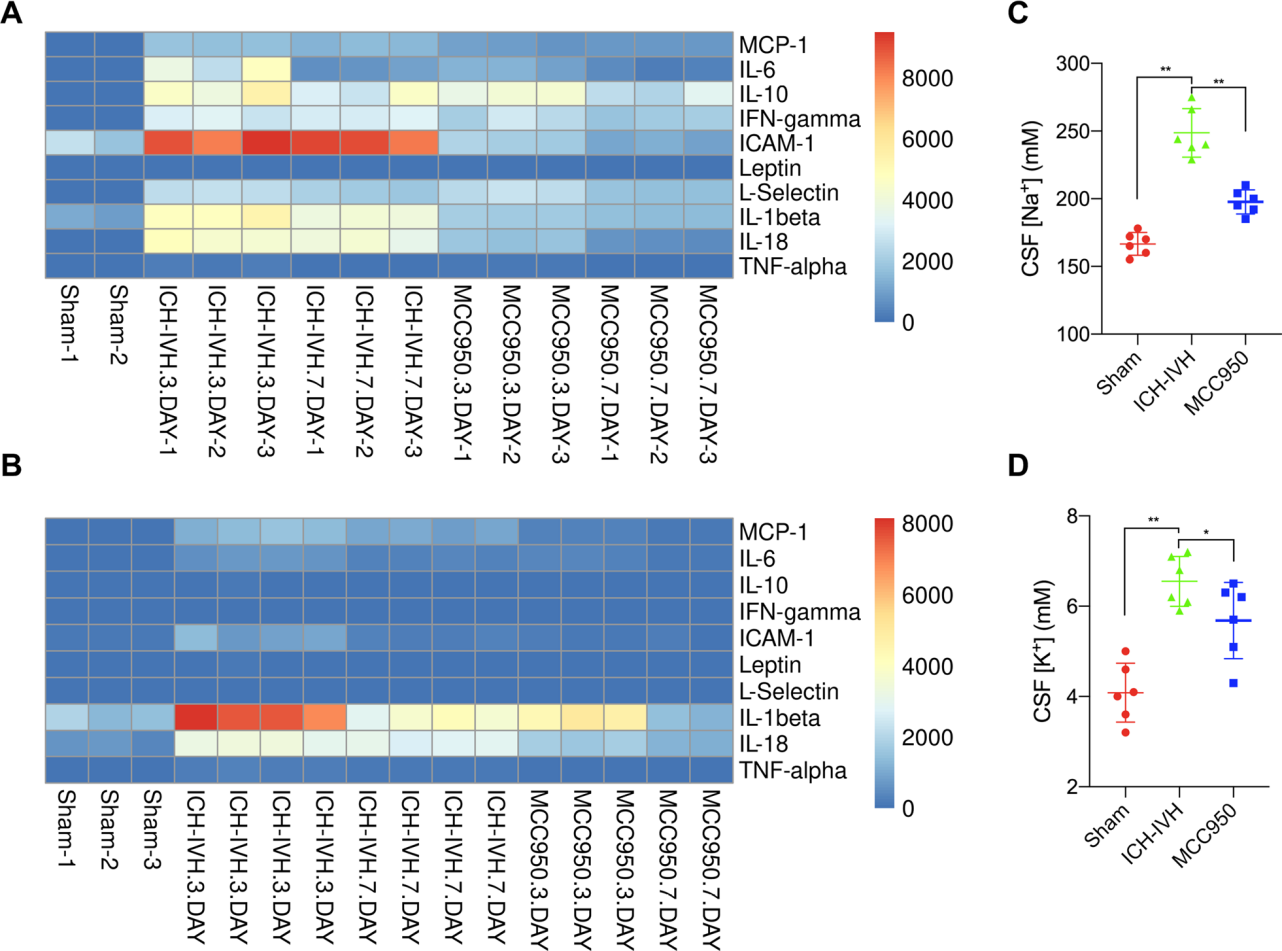
Cytokine expression and ion concentration in the choroid plexus and CSF after ICH-IVH. **A** Heatmap of cytokine assay results of the choroid plexus after ICH-IVH with or without MCC950 treatment. **B** Results of cytokine assays of CSF after ICH-IVH and MCC950 treatment. **C** Na^+^ concentration changes in CSF after ICH-IVH measured with ICP-OES (6 rats/group, one-way ANOVA). **D** K^+^ concentration in CSF after ICH-IVH and MCC950 treatment (6 rats/group, one-way ANOVA). The values are expressed as the means ± SDs; **p < 0.01 and *p < 0.05

On the basis of the results of the transcriptome and proteomic analysis, we aimed to explore NLRP3 function in the choroid plexus after ICH-IVH. The time course of NLRP3 protein expression showed that NLRP3 was upregulated in the choroid plexus after ICH-IVH and peaked at 3 days after ICH-IVH (Fig. 4A). Choroid plexus epithelial cells are the major component of the choroid plexus, which is located in the ventricle systems. Combined with AE2 (a specific protein marker of the choroid plexus) staining results, the choroid plexus was mainly located in the lateral ventricle, which was selected as a sample collection position (Fig. 4B). In addition, we found more NLRP3-positive cells in the choroid plexus after ICH-IVH than in the sham group (Fig. 4C). Western blots used for quantitative analysis showed that the NLRP3 inflammasome and related proteins, IL-1β and caspase-1, were also upregulated after ICH-IVH (Fig. 5E–H). We also used assay kits to measure cytokines in the choroid plexus and CSF, and the reported NLRP3-related cytokines IL-1 and IL-18 presented higher levels in the ICH-IVH group than in the sham group (Fig. 6A, B). These results demonstrated that NLRP3 inflammasome components were activated in choroid plexus epithelial cells after ICH-IVH and peaked at 3 days.

### Treatment with MCC950 or Nlrp3^−/−^ rats reduced CSF secretion, ameliorated hydrocephalus, and improved neurofunction

NLRP3 was selected as the main inflammation target in the acute-phase response and was upregulated in choroid plexus epithelial cells, with a simultaneous increase in CSF secretion. To determine whether NLRP3 was a therapeutic target for adjusting CSF secretion for hydrocephalus in choroid plexus epithelial cells, MCC950 was first selected to inhibit NLRP3, a specific inhibitor of NLRP3. The NLRP3 activator MSU was also used to explore NLRP3’s function in hydrocephalus. In the ICH-IVH rats, MCC950 treatment decreased the lateral ventricle volumes and CSF secretion rates compared with those in the saline injection group (Fig. 5A–C). When NLRP3 was activated again by MSU after MCC950 was used to inhibit NLRP3, the MCC950 + MSU group had larger lateral ventricles and higher CSF secretion rate than the MCC950 group did after ICH-IVH (Fig. 5A–C). In total, the lateral ventricle volumes and CSF secretion rate changed as NLRP3 intervened. These results indicated that NLRP3 is related to CSF hypersecretion-mediated hydrocephalus. At the same time, we evaluated NLRP3-positive cells in the choroid plexus and found that NLRP3-positive cell counts had the same change trend as the CSF secretion rate and lateral ventricular volumes (Fig. 5D). Additionally, western blots were used to quantitatively analyze NLRP3 and related protein expression. The results showed that NLRP3, IL-1β, and Caspase-1 were downregulated after MCC950 treatment in ICH-IVH rats, and MSU treatment intervened in NLRP3-related protein levels in the choroid plexus (Fig. 5E–H). To further explore NLRP3’s contribution to hydrocephalus, we developed Nlrp3^−/−^ rats and found that the Nlrp3^−/−^ group had lower CSF secretion and lateral ventricular volumes than the control group did after ICH-IVH (Fig. 5A–C). NLRP3 staining and quantitative analysis were used to evaluate NLRP3 expression levels, and NLRP3-positive cells were obviously decreased in the Nlrp3^−/−^ group (Fig. 5D). NLRP3, IL-1β and Caspase-1 were also drastically reduced in the Nlrp3^−/−^ group compared with the control group after ICH-IVH (Fig. 5I). Neurofunction (mNSS, number of hindlimb drops, and grip front force) was assessed in different groups, and neurofunction improved in both the MCC950-treated group and the Nlrp3^−/−^ group after ICH-IVH compared with the control (saline) group (Fig. 5J–L). In summary, these results indicated that NLRP3-mediated CSF hypersecretion plays an important role in the occurrence of hydrocephalus after ICH-IVH.

### Inflammatory cytokines are upregulated in the choroid plexus and CSF after hemorrhage

It has been suggested that NLRP3 activation in the choroid plexus contributes to hydrocephalus by upregulating CSF secretion. As such, a cytokine assay was used to evaluate NLRP3-related inflammatory cytokines in the choroid plexus. The choroid plexus assay results indicated that IL-1β, IL-6, IL-10, TNF-α, and IL-18 were upregulated after ICH-IVH, and MCC950 treatment decreased cytokine levels in the choroid plexus (Fig. 6A). CSF is mainly secreted by the choroid plexus, which could reflect the choroid plexus’s function. CSF composition changes have been proven to exist in many kinds of central system diseases. Next, CSF was collected to indicate the cytokine changes. We found that the NLRP3 inflammasome-related cytokines IL-1β and IL-18 were increased in the CSF (Fig. 6B). Moreover, the NLRP3 inflammasome influences CSF secretion. We tested ion changes in CSF, and we also found Na^+^ and K^+^ increased in CSF after ICH-IVH. When inhibiting NLRP3 activation with MCC950 in the choroid plexus decreased CSF secretion and hydrocephalus, the Na^+^ and K^+^ concentrations also decreased in the CSF (Fig. 6C, D). Based on these results, we explored the transmembrane proteins that alter ion transport and water flow, which could be adjusted by NLRP3.

### NKCC1 phosphorylation decreased after ICH-IVH treatment with MCC950 or in Nlrp3^−/−^ rats

**Fig. 7:**
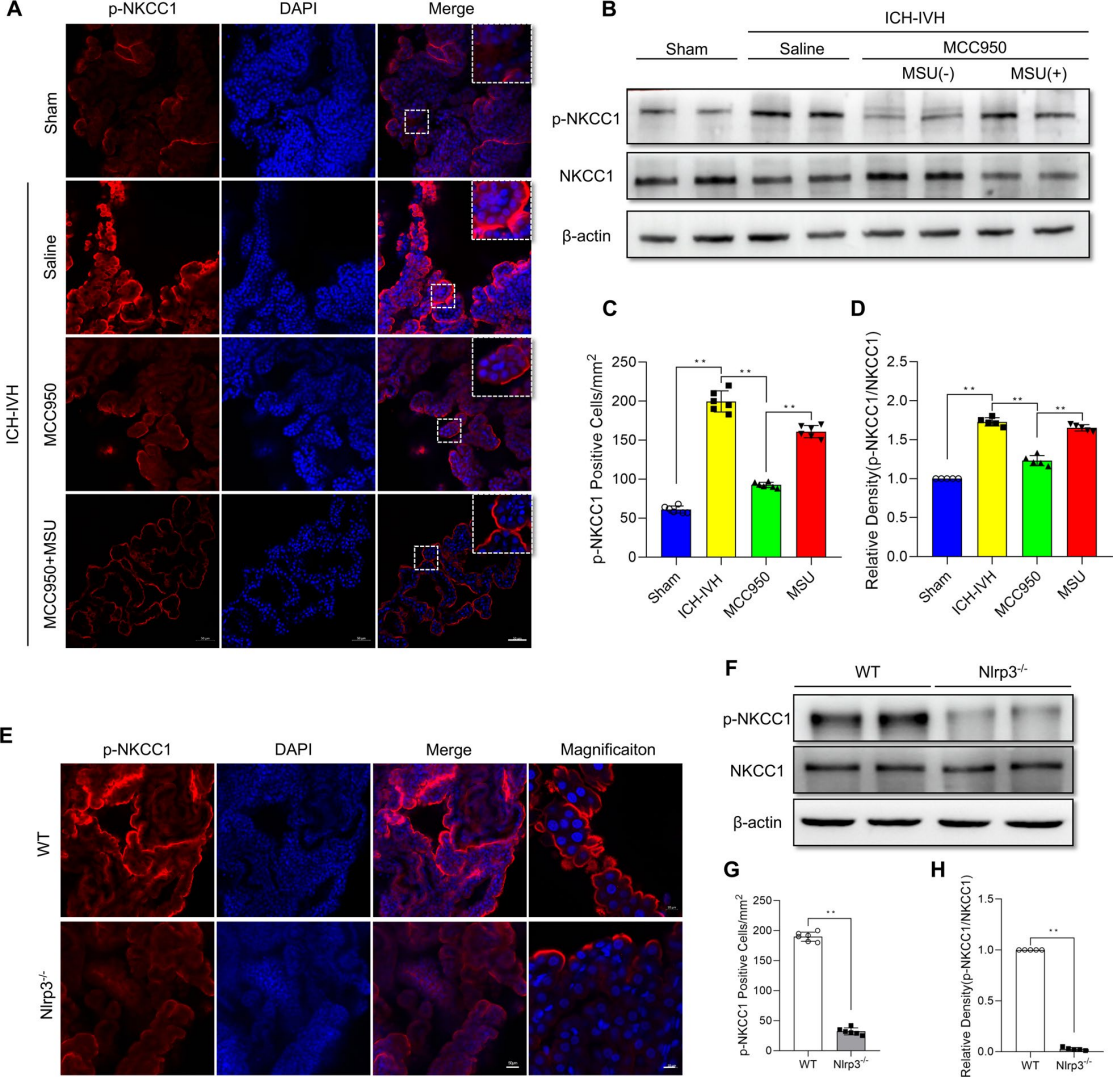
Effects of MCC950 or Nlrp3 knockout on p-NKCC1 protein levels after ICH-IVH and different treatments. **A**, **C** Representative images of immunofluorescence staining for p-NKCC1 (**A**) and p-NKCC1-positive cell statistics (**C**) after inhibition of NLRP3 via MCC950 and reactivation with MSU in the choroid plexus on day 3 (6 rats/group, one-way ANOVA). Bar = 50 μm. **B**, **D** Western blot analysis of p-NKCC1 and NKCC1 in the choroid plexus on day 3 (*n* = 5, 6 rats/group, one-way ANOVA). **E**, **G** p-NKCC1 staining images of the choroid plexus (**E**) and statistical results of p-NKCC1-positive cell counting (**G**) in control and Nlrp3^−/−^ rats after ICH-IVH (6 rats/group, Welch’s two-tailed unpaired t-test). **F**, **H** Quantitative analysis of p-NKCC1 and NKCC1 levels in the choroid plexus in control and Nlrp3^−/−^ rats after ICH-IVH (*n* = 5, 6 rats/group, Welch’s two-tailed unpaired *t*-test). **C**, **D** ICH-IVH indicates ICH-IVH (WT) + saline; MCC950 indicates ICH-IVH (WT) + MCC950; MSU indicates ICH-IVH (WT) + MCC950 + MSU. The values are expressed as the means ± SDs; **p < 0.01; *p < 0.05

NKCC1 is the main transporter that contributes to CSF formation by altering Na^+^, K^+^, and Cl^−^ transport across the membrane, and it has been confirmed that NKCC1 phosphorylation (at residues Thr203, Thr207, and Thr212) could influence its function [38]. First, we found that, at 3 days after ICH-IVH, more p-NKCC1-positive cells were found in the ICH-IVH group than in the sham group, and MCC950 treatment decreased the number of p-NKCC1-positive cells in the choroid plexus. After MSU was used to activate NLRP3 again after MCC950 treatment, the number of p-NKCC1-positive cells increased (Fig. 7A, C). Next, for quantitative analyses of NKCC1 phosphorylation levels, western blots were used. Compared with that of the sham group, the protein expression of p-NKCC1 after ICH-IVH was reduced by MCC950, and MSU treatment intervened in this change (Fig. 7B, D). In Nlrp3^−/−^ rats, NKCC1 phosphorylation was downregulated compared with that in the control group after ICH-IVH according to p-NKCC1 positive counting and quantitative analysis (Fig. 7E–H). Based on these results, we presumed that NKCC1 is related to the NLRP3 inflammasome and that NKCC1 is the downstream molecule of NLRP3. The NLRP3/p-NKCC1 pathway activated CSF hypersecretion in the choroid plexus.

### Treatment with bumetanide reduced NKCC1 phosphorylation and decreased the CSF secretion rate but did not affect NLRP3 expression

**Fig. 8:**
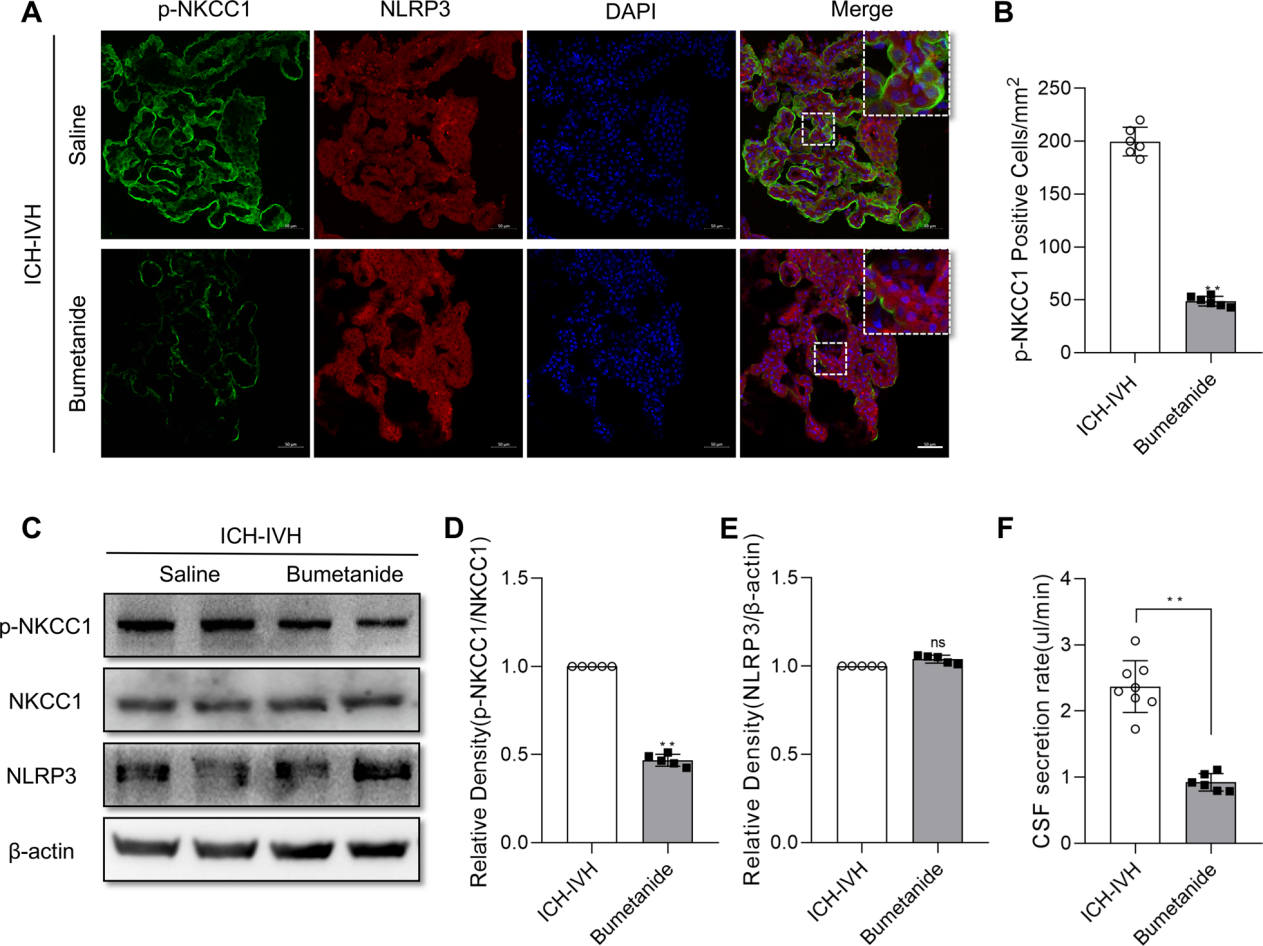
Effects of bumetanide treatment on the choroid plexus after ICH-IVH. **A**, **B** Representative images of immunofluorescence staining for p-NKCC1 (**A**) and p-NKCC1-positive cell statistics (**B**) of the choroid plexus at 3 days following bumetanide treatment (6 rats/group, Welch’s two-tailed unpaired *t*-test). Bar = 50 μm. **C** CSF secretion rates after bumetanide treatment (6–8 rats/group, Welch’s two-tailed unpaired *t*-test). **C**–**E** Western blot analysis of p-NKCC1 and NLRP3 in the choroid plexus at 3 days after bumetanide treatment (*n* = 5, 6 rats/sample, Welch’s two-tailed unpaired *t*-test). **F** CSF secretion rates after bumetanide treatment (6–8 rats/group, Welch’s two-tailed unpaired *t*-test). The values are expressed as the means ± SDs; **p < 0.01; ns, *p* > 0.05

Previous results confirmed that inhibiting NLRP3 using MCC950 or in Nlrp3^−/−^ rats decreased p-NKCC1 and improved hydrocephalus. To prove the function of NKCC1 in CSF secretion, NKCC1 could be adjusted by NLRP3. The NKCC1-specific inhibitor bumetanide reduced the number of p-NKCC1-positive cells in the choroid plexus after ICH-IVH (Fig. 8A, B). In addition, bumetanide reduced the CSF secretion rate after ICH-IVH (Fig. 8F, C), and the protein expression of p-NKCC1 was reduced by bumetanide after ICH-IVH (Fig. 8C, D). However, bumetanide treatment did not reduce NLRP3 protein expression in the choroid plexus after ICH-IVH (Fig. 8C, E). Thus, bumetanide could decrease the CSF secretion rate by inhibiting NKCC1 phosphorylation without influencing NLRP3. These results proved that NKCC1 is the downstream molecule of NLRP3.

### Reduced inflammatory response in primary choroid plexus epithelial cells treated with MCC950 after LPS or lysis-RBC stimulation

**Fig. 9:**
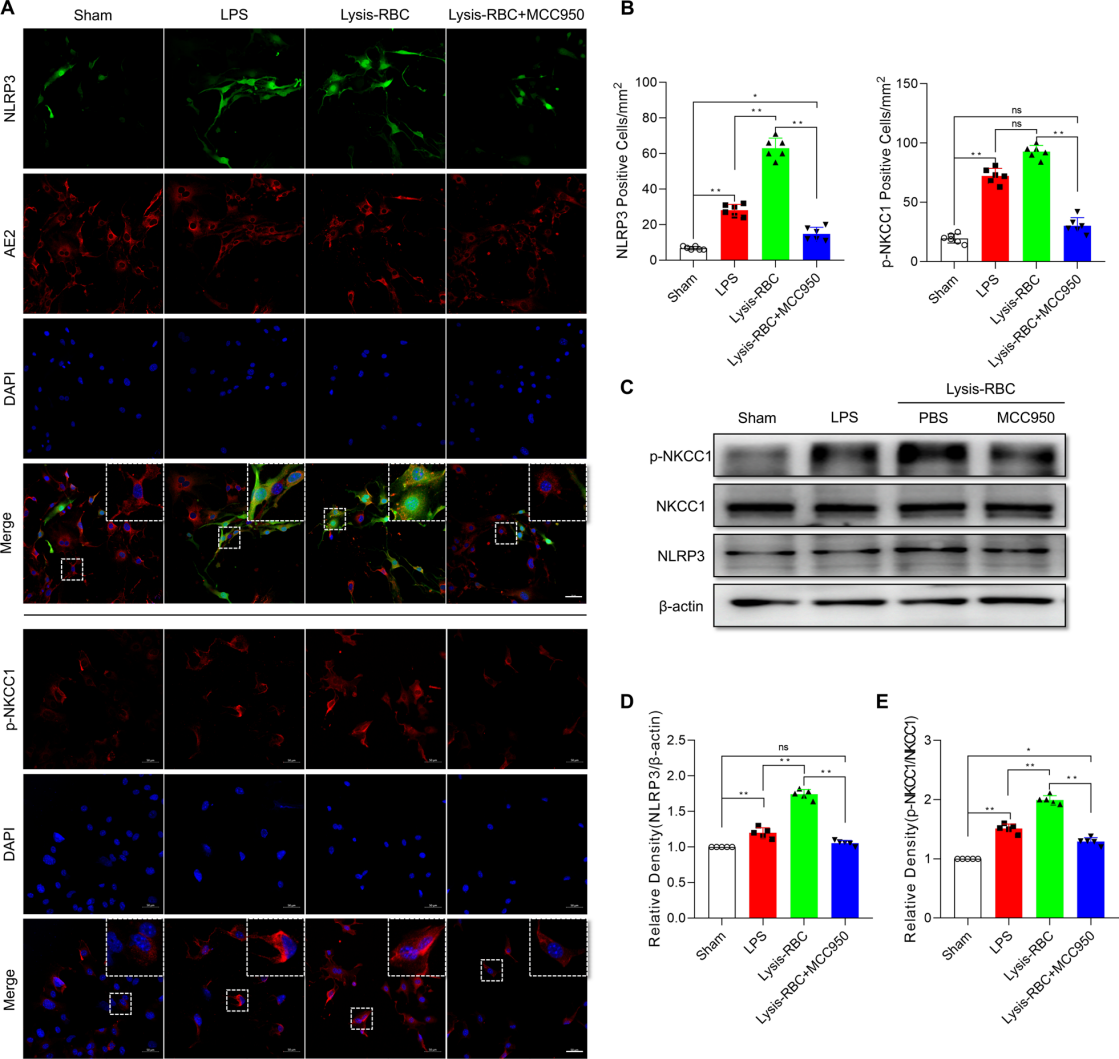
NLRP3/p-NKCC1 pathway activation in primary choroid plexus epithelial cells after LPS or lysis-RBC stimulation. **A** Immunoreactivity of NLRP3 (green) and p-NKCC1 (red, down) as shown by immunofluorescence microscopy in rat primary choroid plexus epithelial cells exposed to LPS or lysis-RBCs and treated with MCC950. Primary choroid plexus epithelial cells were identified and stained with AE2. Nuclei were stained with DAPI. Bar = 50 μm. **B** Morphometric analysis of NLRP3 and p-NKCC1 immunoreactivity in primary choroid plexus epithelial cells exposed to LPS or lysis-RBCs and treated with MCC950 (*n* = 6, one-way ANOVA). **C**–**E** Western blot quantitative analysis of NLRP3 and p-NKCC1 in primary choroid plexus epithelial cells after different treatments (*n* = 5; one-way ANOVA). The values are expressed as the means ± SDs; **p < 0.01 and *p < 0.05, ns, *p* > 0.05

Next, in vitro studies were performed to assess the function of NLRP3 on choroid plexus epithelial cells in an inflammatory state. Lipopolysaccharides are endotoxins derived from the outer leaflet of the outer membrane of Gram-negative bacteria that prime the NLRP3 inflammasome in many kinds of cells [39]. Many kinds of blood metabolites, including lysis-RBCs, could induce neuroinflammation after hemorrhage [1, 40]. Therefore, to imitate in vivo choroid plexus NLRP3 activation, lysis-RBCs separated from arterial blood were used to treat primary choroid plexus epithelial cells. Primary choroid plexus epithelial control cells were cultured and stimulated with 1 μg/ml LPS or 1 μg/ml lysis-RBCs for 12 h, and MCC950 (5 μM) was used to inhibit NLRP3 inflammasome activation in the lysis-RBC-treated group. NLRP3-positive choroid plexus epithelial cells (marked with AE2: red) were measured in different treatment groups, and LPS and lysis-RBC stimulation activated the NLRP3 inflammasome (Fig. 9A) [41]. Our in vivo experimental results showed that p-NKCC1-positive cells were also increased after NLRP3 inflammasome activation. Next, MCC950 treatment decreased NLRP3-positive cells after lysis-RBC treatment, and pNKCC1-positive cells also decreased (Fig. 9B). Furthermore, western blots were used to quantitatively analyze NLRP3 and p-NKCC1 levels in primary choroid plexus epithelial cells (ChP), which showed results similar to those shown before (Fig. 9C–E). In total, the NLRP3/pNKCC1 pathway was activated in ChP after LPS or lysis-RBC stimulation, and MCC950 treatment reduced the activity of the NLRP3/pNKCC1 pathway.

### Na^+^ and K^+^ efflux driving H_2_O outflow after the NLRP3/pNKCC1 pathway is activated in primary choroid plexus epithelial cells

**Fig. 10:**
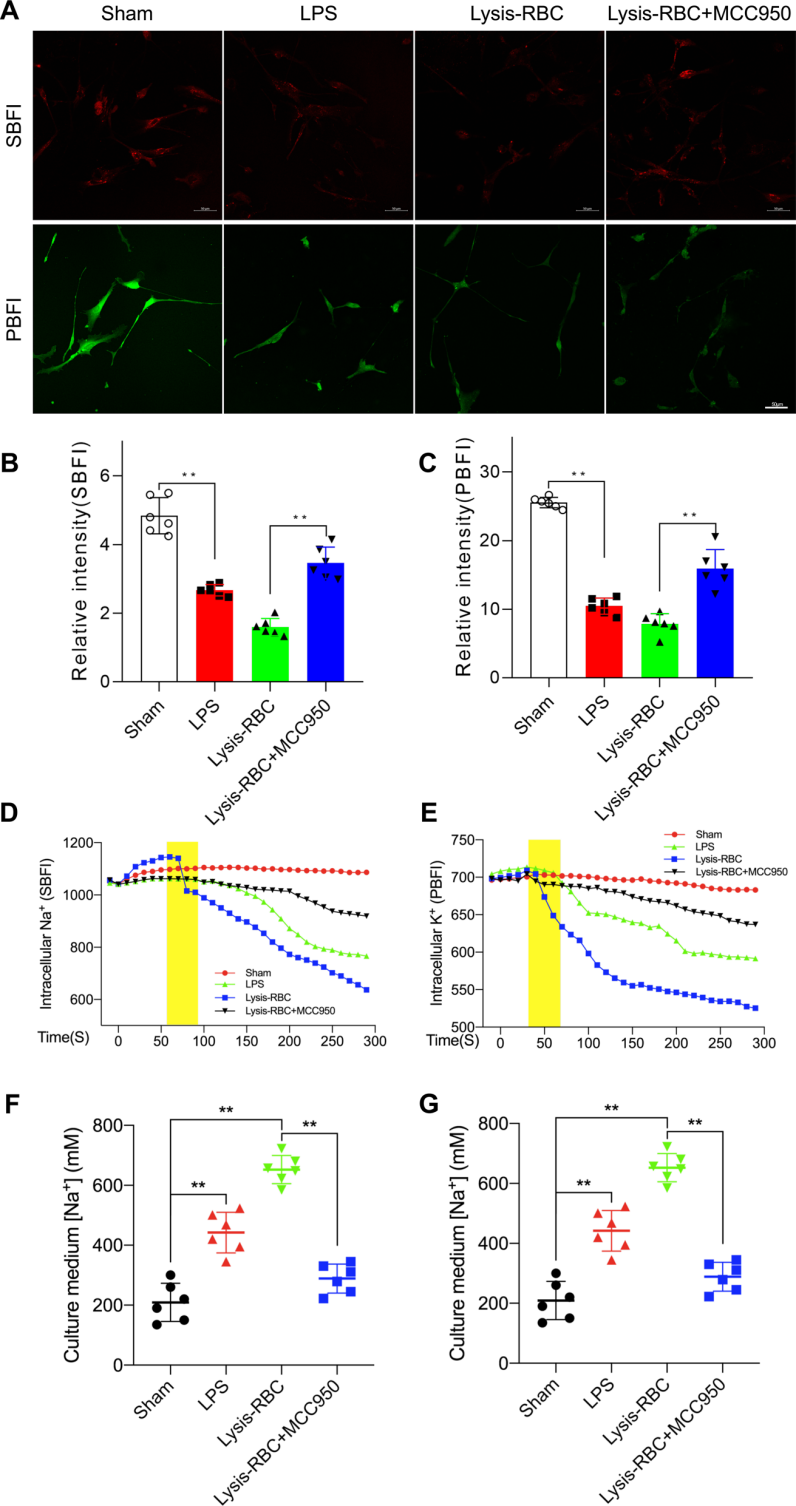
NKCC1-mediated transmembrane cotransport of ions and water after NLRP3 activation. **A** Representative images of Na^+^ (SBFI) and K^+^ (PBFI) after LPS or lysis-RBC exposure and MCC950 treatment in primary choroid plexus epithelial cells. Bar = 50 μm. **B** Statistical results of Na^+^ concentrations in primary choroid plexus epithelial cells according to SBFI staining (*n* = 6, one-way ANOVA). **C** Statistical results of K^+^ concentrations in primary choroid plexus epithelial cells according to PBFI staining (*n* = 6, one-way ANOVA). **D** Time course of Na^+^ concentration changes in primary choroid plexus epithelial cells after the addition of stimulators and MCC950 treatment (*n* = 6). **E** Changes in intracellular K^+^ concentrations over time after different treatments (*n* = 6). **F**–**G** Na^+^ and K^+^ concentrations measured by ICP-OES in culture media after LPS and lysis-RBC stimulation and MCC950 treatment (*n* = 6, one-way ANOVA). The values are expressed as the means ± SDs; **p < 0.01 and *p < 0.05

As reported, NKCC1 is an important transporter located in primary epithelial cells. NKCC1 alters transmembrane ion flow, including Na^+^, K^+^, and Cl^−^, and H_2_O transport across the membrane. Therefore, Na^+^ and K^+^ concentration changes could be used to reflect transmembrane water transport [42]. The CSF detection results proved that Na^+^ and K^+^ increased after hemorrhage, and inhibiting NLRP3 via MCC950 decreased Na^+^ and K^+^ concentrations in CSF. First, we evaluated Na^+^ and K^+^ concentration. SBFI and PBFI were used to measure Na^+^ and K^+^ concentrations in ChP. According to SBFI and PBFI staining images and statistical results, we found that after activation of the NLRP3/pNKCC1 pathway via LPS or lysis-RBCs, the Na^+^ and K^+^ ion concentrations in cells decreased, and using MCC950 to inhibit NLRP3 intervened in the transport of ions across the membrane (Fig. 10A–C). Next, for SBFI- and PBFI-loaded ChP, we monitored Na^+^ and K^+^ dynamics for 300 s when LPS and lysis-RBCs were added to the media. Intracellular Na^+^ and K^+^ decreased immediately after stimulators were added compared with that in the sham group, and the addition of MCC950 weakened ion and water outflow (Fig. 10D, E). Then, we measured Na^+^ and K^+^ in the media for reevaluation via ICP-OES and found that the Na^+^ and K^+^ concentrations in the media were elevated in both the LPS- and lysis-RBC-treated groups, and inhibiting NLRP3/pNKCC1 decreased the ion concentration in the medium (Fig. 10F, G). In summary, NLRP3/p-NKCC1 induced the outflow of water from choroid plexus epithelial cells.

## Discussion

**Fig. 11:**
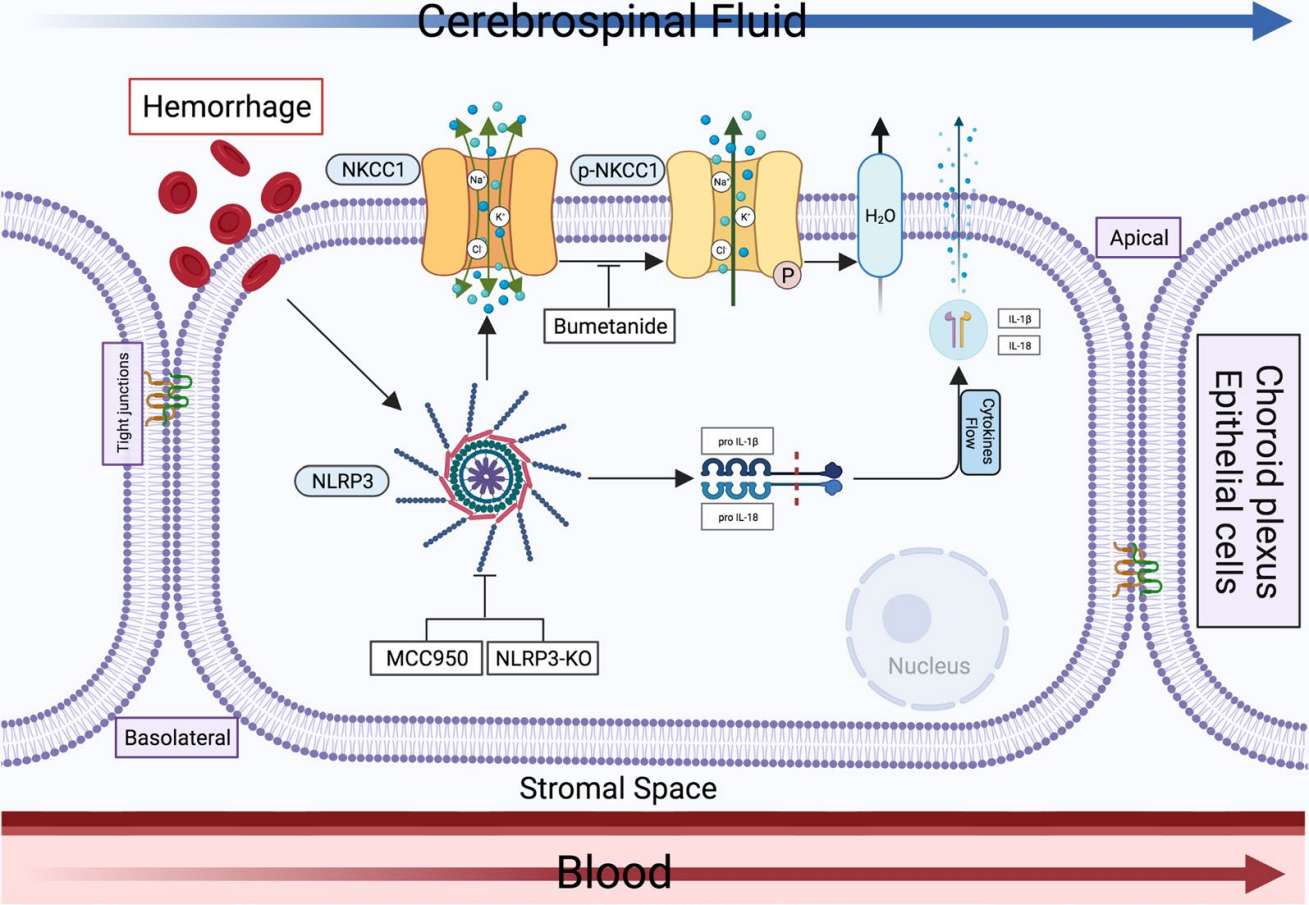
Schematic summary of the present study. Schematic mechanism through which NLRP3/p-NKCC1-activated CSF hypersecretion contributes to hydrocephalus in the choroid plexus after hemorrhage. After hemorrhage, the NLRP3 inflammasome was activated, which could secrete IL-1β and IL-18 into the CSF. NLRP3 altered NKCC1 phosphorylation to influence transmembrane water transport. NLRP3/p-NKCC1-mediated CSF hypersecretion contributes to hydrocephalus

We demonstrated that activation of the NLRP3 inflammasome contributed to hydrocephalus by mediating CSF hypersecretion in the choroid plexus after ICH-IVH. Furthermore, the NLRP3 inflammasome influenced CSF secretion by controlling NKCC1 phosphorylation in the choroid plexus, and NKCC1 affected hydrocephalus by transporting ions across the membrane together with water. Combined, these results suggest that the NLRP3 inflammasome contributes to the pathogenesis of hydrocephalus after ICH-IVH (Fig. 11).

Hydrocephalus is a severe complication after ICH, especially with ventricular extension [43, 44]. Most studies on hydrocephalus after hemorrhage have been based on the IVH model and have not been able to simulate the clinical pathogenesis of hydrocephalus after hemorrhage in infants or adults [45]. As revealed by our previous study, hydrocephalus and brain tissue injury were found to be more serious in a rat model of ICH-IVH than in an IVH model, which thus conforms more closely to the results of a clinical model [9]. Therefore, we used the ICH-IVH model to explore the pathogenesis of hydrocephalus in this study.

It is a commonly held view that hydrocephalus after hemorrhage is caused by abnormalities in the CSF drainage pathway, particularly related to the cerebral aqueduct, fourth ventricular outlets, ependymal epithelium damage, arachnoid villi or granulations or choroid plexus inflammation. Few animal studies have investigated the cerebral aqueduct or the outlets of the fourth ventricle. Arachnoid granulations are considered sites of absorption. The presence and morphology of arachnoid granulations are different between humans and animals [46]. However, arachnoid granulations are not completely developed in infants with hydrocephalus [47, 48]. Nerve tissue with lymphatic vessels has been widely reported [49–51]. It is accepted that a considerable amount of CSF flows into lymphatic vessels and that lymphatic outflow may result in hydrocephalus [52]. Ependymal cilium beating generates flow of the CSF within the brain cavities and aids in maintaining the patency of the ventricular system [53, 54]. Clinical and preclinical data suggest that the loss of developing and mature ciliated epithelial cells contributes to the development of hydrocephalus after hemorrhage [55–57]. All these studies focused on the outflow dysfunction of CSF. The choroid plexus is located at the base of each of the four ventricles. Regarding the production of CSF in the choroid plexus, few relevant studies have been conducted. Nuclear factor κB (NF-κB) signaling is activated by CSF barrier cells of the choroid plexus and ependymal lining after IVH [58]. Moreover, systemic inflammation stimulates TLRs in the choroid plexus, which may lead to disturbances in choroid plexus barrier function [59]. A recent study proved that TLR4-dependent inflammation leading to CSF hypersecretion plays a role in the development of hydrocephalus after hemorrhage [11]. It has been reported that NLRP3 mediates many kinds of nervous system injuries after ICH, subarachnoid hemorrhage or traumatic brain injury [22, 60], but there are no studies about the NLRP3 inflammasome in hydrocephalus. In addition, transcriptome and proteomics analyses of the choroid plexus proved that the inflammatory response, including NLRP3 inflammasome activation, was activated. It has also been proven through single-cell RNA-seq that the NLRP3 inflammasome is expressed in epithelial cells. Based on these findings, we investigated the characteristics of the NLRP3 inflammasome and the choroid plexus and proved that NLRP3 contributes to hydrocephalus by increasing CSF secretion in the choroid plexus. Current CSF secretion methods indirect for measuring CSF formation in vivo include tracer dilution, MRI and others. CSF formation was measured using the indirect tracer dilution method with blue dextran, but the limitation cannot be synchronized with pharmacological manipulations [61]. A single method was used in this study to measure CSF secretion, but other approaches should also be explored. Furthermore, blood components are complex, which makes identifying the specific components that activate the NLRP3 inflammasome in the choroid plexus unclear. Many factors, such as iron, hemoglobin and transforming growth factor-β1, contribute to hydrocephalus after hemorrhage [9, 62, 63]. In in vitro studies, only lysis-RBCs were used to stimulate primary choroid plexus epithelial cells, which activated the NLRP3 inflammasome. In future studies, we aim to explore what activates the NLRP3 inflammasome after ICH-IVH in the choroid plexus.

After we discovered that the NLRP3 inflammasome became activated after ICH-IVH in the choroid plexus, we attempted to elucidate the molecular mechanisms occurring between the NLRP3 inflammasome and CSF formation. Approximately 500 ml of fluid is produced by the choroid plexus in the mammalian brain. CSF production is assumed to take place by the transport of osmotically active ions, the passive movement of water and the water channel aquaporin. NKCC1, K^+^/Cl^−^ cotransporters (KCCs), Na^+^-coupled bicarbonate transporters, and Na^+^/K^+^-ATPase are the main ion transporters that adapt to the formation of CSF, and NKCC1 plays an important role among these transporters [13, 14, 64–68]. However, CSF production decreased by only 20% in AQP1 knockout mice [69]. AQP4 also contributes to CSF formation, but its function is also limited [70]. NKCC1 is located at the luminal membrane and indicates its unique outward transport direction, which mediates the cotransport of water by ion transport across the membrane in an inflammatory state. Among the concentrations of Na^+^, K^+^, and Cl^−^ altered by NKCC1, we detected Na^+^ and K^+^ concentrations in primary choroid plexus epithelial cells. The main regulatory molecule NKCC1 was selected in this study, and it was briefly proven that NKCC1 is a molecular component in NLRP3-mediated CSF hypersecretion. Interestingly, the ion concentration results confirmed our previous results; however, Na^+^ and K^+^ showed different change trends, and the degree of Na^+^ outflow was more severe than that of K^+^ outflow. K^+^ efflux and inflammation stimulators (LPS or lysis-RBC) can activate the NLRP3 inflammasome [71, 72]. After the NLRP3/p-NKCC1 pathway is activated by stimulators in the choroid plexus, p-NKCC1 mediates Na^+^ and K^+^ outflow, and K^+^ efflux activates the NLRP3 inflammasome, which results in a positive feedback cascade amplification effect. Due to the limited evidence, further studies are still needed to clarify the mechanism of NLRP3-mediated CSF hypersecretion after ICH-IVH.

In this study, we observed that ICH-IVH induced CSF hypersecretion by activating the NLRP3 inflammasome, and the ion cotransporter NKCC1 regulating CSF secretion also participated in this process. In addition, we found blood–CSF barrier disruption and lipid droplet (LD) formation in the choroid plexus by accident.

The most widely recognized role of the choroid plexus is in the formation of the blood–CSF barrier, whereby it serves as a controller of the internal CNS microenvironment [73]. Blood–CSF barrier integrity is impaired in the pathology of many common CNS disorders, such as Alzheimer's disease, Parkinson's disease and stroke [74]. Neuroinflammation is a natural response to CNS injury and disease during blood–CSF barrier dysfunction [75]. We found that after blood–CSF barrier dysfunction due to ICH-IVH, inhibition of the NLRP3 inflammasome could improve the integrity of the blood–CSF barrier. Nonetheless, the relationship between the integrity of the blood–CSF barrier and the NLRP3 inflammasome requires further exploration. LDs are major lipid storage organelles of eukaryotic cells and are central players in anti-infection processes [76]. Further studies will focus on what components in the blood contribute to NLRP3 inflammasome activation, blood–CSF barrier disruption and LD formation and the relationship between them and hydrocephalus. Although we identified a new therapeutic target for hydrocephalus after hemorrhage, the pathogenesis of hydrocephalus requires further exploration for a complete understanding.

## Conclusion

Our results showed that activation of NLRP3 in the choroid plexus contributed to increased CSF secretion and aggravated hydrocephalus after ICH-IVH, and NLRP3 impacted CSF secretion by regulating the NKCC1 phosphorylation level. In addition, NKCC1 phosphorylation mediated more coupling of ion (Na^+^ and K^+^) efflux to transmembrane water transport in primary choroid plexus epithelial cells after ICH-IVH, which also contributed to aggravated hydrocephalus. This study provides evidence that inhibiting NLRP3 may be a potential therapeutic approach for preventing hydrocephalus after ICH-IVH.

## Supplementary Information

**Additional file 1.** Statistical Results in Figures.

## Data Availability

All data generated or analyzed during this study are included in this published article. The datasets used and/or analyzed during the current study are available from the corresponding author on reasonable request.

## Abbreviations

ICH: Intracerebral hemorrhage
CSF: Cerebrospinal fluid
TLR4: Toll-like receptor 4
NKCC1: Na^+^/K^+^/2Cl^−^ cotransporter
ICH-IVH: Intracerebral hemorrhage with ventricular extension
ARRIVE: Animal Research: Reporting of In Vivo Experiments
mNSS: Modified Neurological Severity Score
FPKM: Fragments per kilobase per million
IVH: Intraventricular hemorrhage
MRI: Magnetic resonance imaging
ChP: Primary choroid plexus epithelial cells
