# Supplementary material for: NLRP3 inflammasome-mediated choroid plexus hypersecretion contributes to hydrocephalus after intraventricular hemorrhage via phosphorylated NKCC1 channels
Source: J Neuroinflammation. 2022 Jun 21;19:163. doi: 10.1186/s12974-022-02530-x (PMC9210649; doi:10.1186/s12974-022-02530-x)
Supplement: Supplementary file 1 — Additional file 1. Statistical Results in Figures. [file 12974_2022_2530_MOESM1_ESM.pdf]

Figure 2

2B: 3day sham vs. IVH P<0.0001; sham vs. ICH-IVH P<0.0001; IVH vs. ICH-IVH P=0.04.

7day sham vs. IVH P<0.0001; sham vs. ICH-IVH P<0.0001; IVH vs. ICH-IVH P=0.015.

2C: 3day sham vs. IVH P=0.0001; sham vs. ICH-IVH P<0.0001; IVH vs. ICH-IVH P=0.024.

7day sham vs. IVH P=0.0002; sham vs. ICH-IVH P=0.0008; IVH vs. ICH-IVH P=0.5404.

2E: 0day sham vs. IVH P=0.89; sham vs. ICH-IVH P=0.99; IVH vs. ICH-IVH P=0.89.

1day sham vs. IVH P=0.1514; sham vs. ICH-IVH P=0.0454; IVH vs. ICH-IVH P=0.85.

3day sham vs. IVH P=0.0383; sham vs. ICH-IVH P=0.0006; IVH vs. ICH-IVH P=0.015.

7day sham vs. IVH P=0.0032; sham vs. ICH-IVH P=0.0002; IVH vs. ICH-IVH P=0.0128.

2F: 0day sham vs. IVH P=0.42; sham vs. ICH-IVH P=0.71; IVH vs. ICH-IVH P=0.87.

1day sham vs. IVH P<0.0001; sham vs. ICH-IVH P<0.0001; IVH vs. ICH-IVH P=0.02.

3day sham vs. IVH P<0.0001; sham vs. ICH-IVH P<0.0001; IVH vs. ICH-IVH P=0.03.

7day sham vs. IVH P<0.0001; sham vs. ICH-IVH P<0.0001; IVH vs. ICH-IVH P=0.91.

2G: 0day sham vs. IVH P=0.89; sham vs. ICH-IVH P=0.89; IVH vs. ICH-IVH P=0.65.

1day sham vs. IVH P<0.0001; sham vs. ICH-IVH P<0.0001; IVH vs. ICH-IVH P=0.97.

3day sham vs. IVH P<0.0001; sham vs. ICH-IVH P<0.0001; IVH vs. ICH-IVH P=0.46.

7day sham vs. IVH P<0.0001; sham vs. ICH-IVH P<0.0001; IVH vs. ICH-IVH P=0.97.

Figure 4

4A: sham vs. 1day P=0.028; sham vs. 3day P<0.0001; sham vs. 7day P=0.048; 1day vs. 3day P=0.022; 3day vs. 7day P=0.0017;

Figure 5

5B: ICH-IVH vs. MCC950 P=0.0096; ICH-IVH vs. $NLRP3^{-/-}$ P=0.0001; MCC950 vs. MSU P=0.032.

5C: ICH-IVH vs. MCC950 P=0.0038; ICH-IVH vs. $NLRP3^{-/-}$ P<0.0001; MCC950 vs. MSU P=0.015.

5F: sham vs. ICH-IVH P<0.0001; ICH-IVH vs. MCC950 P<<0.0001; MCC950 vs. MSU P=0.032.

5G: sham vs. ICH-IVH P<0.0001; ICH-IVH vs. MCC950 P=0.0002; MCC950 vs. MSU P=0.033.

5H: sham vs. ICH-IVH P<0.0001; ICH-IVH vs. MCC950 P<0.0001; MCC950 vs. MSU P=0.018.

5I: WT vs. $NLRP3^{-/-}$: IL-1beta P<0.0001; NLRP3 P<0.0001; Caspase-1 P<0.0001.

5J: ICH-IVH vs. MCC950 P=0.0002; ICH-IVH vs. $NLRP3^{-/-}$ P<0.0001; MCC950 vs. MSU P=0.002.

5K: ICH-IVH vs. MCC950 P=0.0095; ICH-IVH vs. NLRP3$^{-/-}$ P<0.0001; MCC950 vs. MSU P=0.035.

5L: ICH-IVH vs. MCC950 P<0.0001; ICH-IVH vs. NLRP3$^{-/-}$ P<0.0001; MCC950 vs. MSU P=0.0013.

Figure 6

6C: sham vs. ICH-IVH P<0.0001; ICH-IVH vs. MCC950 P<0.0001.

6D: sham vs. ICH-IVH P<0.0001; ICH-IVH vs. MCC950 P=0.032.

Figure 7

7C: sham vs. ICH-IVH P<0.0001; ICH-IVH vs. MCC950 P<<0.0001; MCC950 vs. MSU P<0.0001.

7D: sham vs. ICH-IVH P<0.0001; ICH-IVH vs. MCC950 P<<0.0001; MCC950 vs. MSU P=0.0003.

7G: WT vs. NLRP3$^{-/-}$ P<0.0001.

7H: WT vs. NLRP3$^{-/-}$ P<0.0001.

Figure 8

8B: Saline vs. Bumetanide P<0.001.

8D: Saline vs. Bumetanide P<0.001.

8E: Saline vs. Bumetanide P=0.21.

8F: Saline vs. Bumetanide P<0.001.

Figure 9

9B: NLRP3: sham vs. LPS P<0.0001; sham vs. Lysis-RBC+MCC950 P=0.024; LPS vs. Lysis-RBC P=0.009; Lysis-RBC vs. Lysis-RBC+MCC950 P<0.0001

p-NKCC1: sham vs. LPS P<0.0001; sham vs. Lysis-RBC+MCC950 P=0.18; LPS vs. Lysis-RBC P=0.105; Lysis-RBC vs. Lysis-RBC+MCC950 P<0.0001.

9D: sham vs. LPS P=0.0042; sham vs. Lysis-RBC+MCC950 P=0.55; LPS vs. Lysis-RBC P=0.001; Lysis-RBC vs. Lysis-RBC+MCC950 P<0.0001.

9E: sham vs. LPS P<0.0001; sham vs. Lysis-RBC+MCC950 P=0.035; LPS vs. Lysis-RBC P=0.007; Lysis-RBC vs. Lysis-RBC+MCC950 P<0.0001.

Figure 10

10B: sham vs. LPS P=0.0025; Lysis-RBC vs. Lysis-RBC+MCC950 P=0.0058.

10C: sham vs. LPS P=0.0003; Lysis-RBC vs. Lysis-RBC+MCC950 P=0.0092.

10F: sham vs. LPS P<0.0001; sham vs. Lysis-RBC P<0.0001; Lysis-RBC vs. Lysis-RBC+MCC950 P<0.0001.

10G: sham vs. LPS P=0.0001; sham vs. Lysis-RBC P<0.0001; Lysis-RBC vs. Lysis-RBC+MCC950 P<0.0001.
